# Segmentectomy or Wedge Resection in Stage IA Lung Squamous Cell Carcinoma and Adenocarcinoma?

**DOI:** 10.7150/jca.49683

**Published:** 2021-01-18

**Authors:** Guoshu Li, Shuanshuan Xie, Feng Hu, Min Tan, Lihong Fan, Changhui Wang

**Affiliations:** 1 Department of Respiratory Medicine, Shanghai Tenth People's Hospital, Tongji University School of Medicine, Shanghai 200072, China.; 2 Tongren Hospital, Shanghai Jiao Tong University School of Medicine, 1111 XianXia Road, Shanghai 200336, China.

**Keywords:** SEER, segmentectomy, wedge resection, non-small cell lung cancer, survival

## Abstract

**Objectives:** We performed this study to compare survival outcomes of segmentectomy (SG) and wedge resection (WR) in stage IA lung squamous cell carcinoma (SQCC) and lung adenocarcinoma (AD).

**Methods:** Using the Surveillance, Epidemiology, and End Results registry (SEER), we identified 1529 and 4070 patients with stage IA SQCC and AD, respectively, who had complete clinical information between 2004 and 2015. We used Kaplan-Meier analysis to determine the propensity score for patients with limited resection based on the preoperative characteristics of patients. Lung cancer-specific survival (LCSS) was compared in patients treated with WR and SG after adjusting, stratifying, or matching lung cancer patients according to propensity score.

**Results:** Kaplan-Meier analysis demonstrated that there was a statistically significant difference in survival curves (log rank *P*=0.01) for patients with stage IA SQCC between SG and WR. But there was no statistically significant difference in survival curves (log rank *P*>0.05) in patients with stage IA AD between the two limited resections. Compared with the WR, The hazard ratios (95% confidence intervals) of SG were 0.689 (0.519-0.914) and 0.896 (0.752-1.067) in patients with stage IA SQCC and AD, respectively.

**Conclusion:** This study suggests that SG can yield superior survival outcome compared with WR in patients with stage IA SQCC. However, the survival outcomes of SG and WR are generally equivalent in patients with stage IA AD.

## Introduction

Lung cancer is a serious threat to human health, and its incidence has risen rapidly in recent years. Patients with advanced non-small cell lung cancer (NSCLC) have a poor prognosis, but those with stage IA NSCLC have relatively good long-term outcomes after appropriate treatment 1. Some studies have reported that the 5-year overall survival rate of patients with stage IA NSCLC after surgery is more than 70%, and the prognosis is even better for patients with tumor sizes ≤2 cm 2-5. Although most patients with NSCLC are diagnosed at an advanced stage, 10%-15% of them are diagnosed with stage IA NSCLC 1. Moreover, with the introduction of high-resolution computed tomography (CT) and low-dose helical CT screening for lung cancer, the number of patients diagnosed with early-stage lung cancer has increased 6. Surgery is one of the most important treatment methods for stage IA NSCLC. Currently, lobectomy remains the standard surgical treatment for patients with NSCLC. However, limited resection is commonly used to treat patients who are unable to undergo total lobectomy due to older age, severe impairment of lung function, or other comorbidities 7. In recent years, many studies have shown that the survival rate of patients with stage IA NSCLC who undergo limited resection is similar to that of patients who undergo lobectomy 8-13. In addition, patients who undergo limited resection have less resected lung tissue and greater retention of lung function than those who undergo conventional lobectomy, which provides an opportunity for subsequent surgery if a second primary lung cancer occurs 14. Therefore, limited resection has become an important treatment for patients with stage IA NSCLC.

Limited resection methods include wedge resection (WR) and segmentectomy (SG). SG, as an anatomic resection, has been thought as a preferred approach compared to WR in patients with stage IA NSCLC 15. However, due to the quality of life in patients receiving SG was not as good as patients undergoing WR 16, 17, the option of WR or SG should be more cautious for patients. Previous studies have compared survival rates between the two types of limited resection primarily based on variables such as tumor size, differentiation grade, age, and so on, but less on the pathology subtypes of NSCLC. This study was to compare survival outcomes of WR and SG by comparing with lung cancer-specific survival (LCSS) in patients with stage IA lung squamous cell carcinoma (SQCC) or lung adenocarcinoma (AD), the major pathology subtypes of NSCLC.

## Methods

### Data source

This retrospective study was conducted to assess the relationship between two types of limited resection and the survival rate in patients with IA stage SQCC or AD, using data from the Surveillance, Epidemiology, and End Results (SEER) database. The SEER project is maintained by the National Cancer Institute in the United States (US). SEER includes a population-based cancer registry, established in 1973, which accounts for approximately 10% of the US population 18.

### Study population

**Figure 1:**
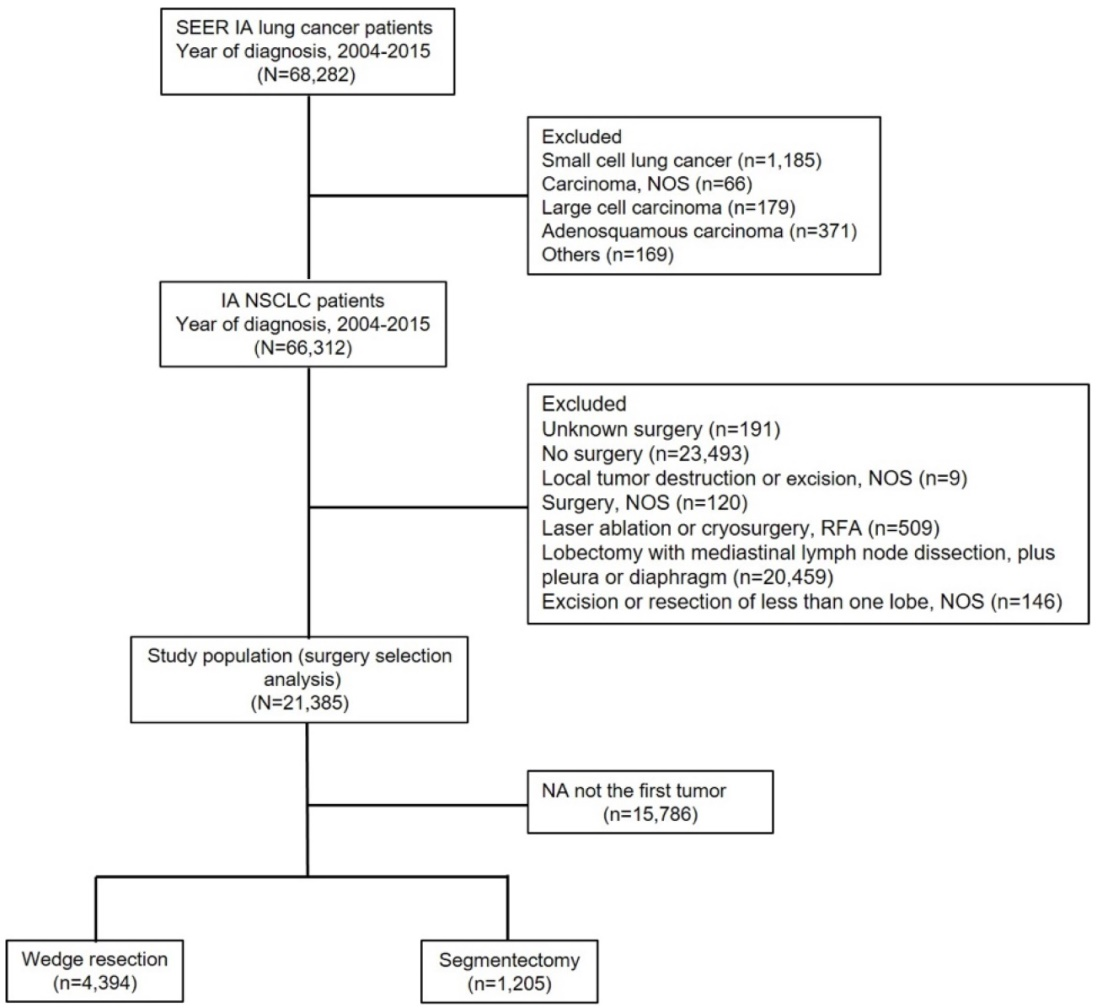
** Study flow diagram.** Abbreviations: NSCLC, non-small cell lung cancer; NOS, not otherwise specified; RFA, radiofrequency ablation.

We limited the cohort to patients with stage IA SQCC or AD (≤3 cm in tumor size) diagnosed between 2004 and 2015. All included patients underwent WR or SG; the complete information of all included patients was available in the SEER database. Figure 1 shows a flow chart of the literature search for analyses based on the SEER database. Initially, 68,282 patients with IA stage lung cancer were identified. A total 66,312 patients with NSCLC were included, after excluding the following patients: 1,185 patients with small cell lung cancer, 66 with carcinoma not otherwise specified (NOS), 179 with large cell carcinoma, 371 with adenosquamous carcinoma and 169 patients with other cancers. We included 21,385 patients with NSCLC who were treated surgically, after excluding the following patients: 191 patients who underwent unknown surgery; 23,493 with no surgery; 9 with local tumor destruction or excision NOS; 120 with surgery NOS; 509 with laser ablation, cryosurgery, or radiofrequency ablation; 20,459 who underwent lobectomy with mediastinal lymph node dissection, plus pleura or diaphragm; and 146 patients with excision or resection of less than one lobe NOS. Finally, a total of 5,599 patients with stage IA SQCC or AD who were treated with limited resection were included in the analysis, after excluding 15,786 patients with NA not the first tumor.

### Clinicopathological data

According to histologic type, histologic codes were classified as follows: (1) SQCC: 8052, 8070-8075, 8083, 8084, 8123, (2) AD: 8244, 8245, 8250-8255, 8260, 8290, 8310, 8323, 8333, 8480, 8481, 8490, 8507, 8550, 8570, 8571, 8574, and 8576. Based on information of the SEER site-specific surgical variables, all 5,599 included patients with stage IA SQCC or AD were classified as having undergone WR (SEER surgical code 21) or SG (SEER surgical code 22).

### Statistical analyses

Classification variables were compared using the Pearson's chi-squared test. Kaplan-Meier analysis and the log-rank test were used to compare survival between the WR group and the SG group. Propensity score methods were used to control the potential differences in baseline characteristics of the included patients. Cox regression was performed to assess whether the baseline covariates of the two groups were balanced after adjusting for the estimated propensity scores. Statistical significance was set at a two-tailed *P* value <0.05. Data were analyzed using IBM SPSS version 20.0 (IBM Corp, Armonk, NY, USA).

## Results

### Study cohort characteristics

We identified 5,599 limited resection patients with stage IA SQCC or AD, of whom 4,394 (78.5%) and 1,205 (21.5%) underwent WR and SG, respectively, as a primary treatment from 2004 to 2015. Table 1 shows the baseline characteristics of all patients. Kaplan-Meier analyses demonstrated that there were no statistically significant differences in LCSS with regard to tumor location (*P=*0.996), marital status (*P*=0.482), laterality (*P*=0.854) and high school education (*P=*0.079) between the two groups. However, significant differences in LCSS were found with respect to age (*P*<0.001), sex (*P*<0.001), tumor size (*P*<0.001), differentiation grade (*P*<0.001), histologic type (*P*<0.001), radiotherapy (*P*<0.001), and chemotherapy (*P*<0.001) (Table 1).

Subsequent analysis using a Cox model, which included above seven significant covariates, showed that there were no statistically significant differences for histologic type (*P*=0.263) in patients with stage IA SQCC or AD. However, there were statistically significant differences for age (*P*<0.001), sex (*P*<0.001), tumor size (*P*<0.001), differentiation grade (*P*<0.001), radiotherapy (*P*<0.001), chemotherapy (*P*<0.001), and resection (*P*=0.013) (Table 2). These outcomes demonstrate that the prognoses of patients with stage IA SQCC or AD are related to the followed factors: age, sex, tumor size, differentiation grade, radiotherapy, chemotherapy, and limited resection, but are not related to these factors: tumor location, marital status and laterality.

Table 3 shows the hazard ratio (HR) and 95% confidence interval (CI) between the WR and SG. Compared with the WR group, the HR (95%CI,* P*) of the SG group were 0.823 (0.710-0.955, *P*=0.01) in total patients. The HR (95%CI, *P*) of the SG group were 0.689 (0.519-0.914, *P*=0.01), 0.896 (0.752-1.067, *P*=0.217) comparing with the WR group in patients with SQCC and AD, respectively. These outcomes demonstrate that the comparative results of WR and SG are different in pathology subtypes of NSCLC: the SG yields better survival outcome than the WR in patients with stage IA SQCC, but the survival outcomes of SG and WR are generally equivalent in patients with stage IA AD.

### Comparison of survival curves between WR and SG

**Figure 2:**
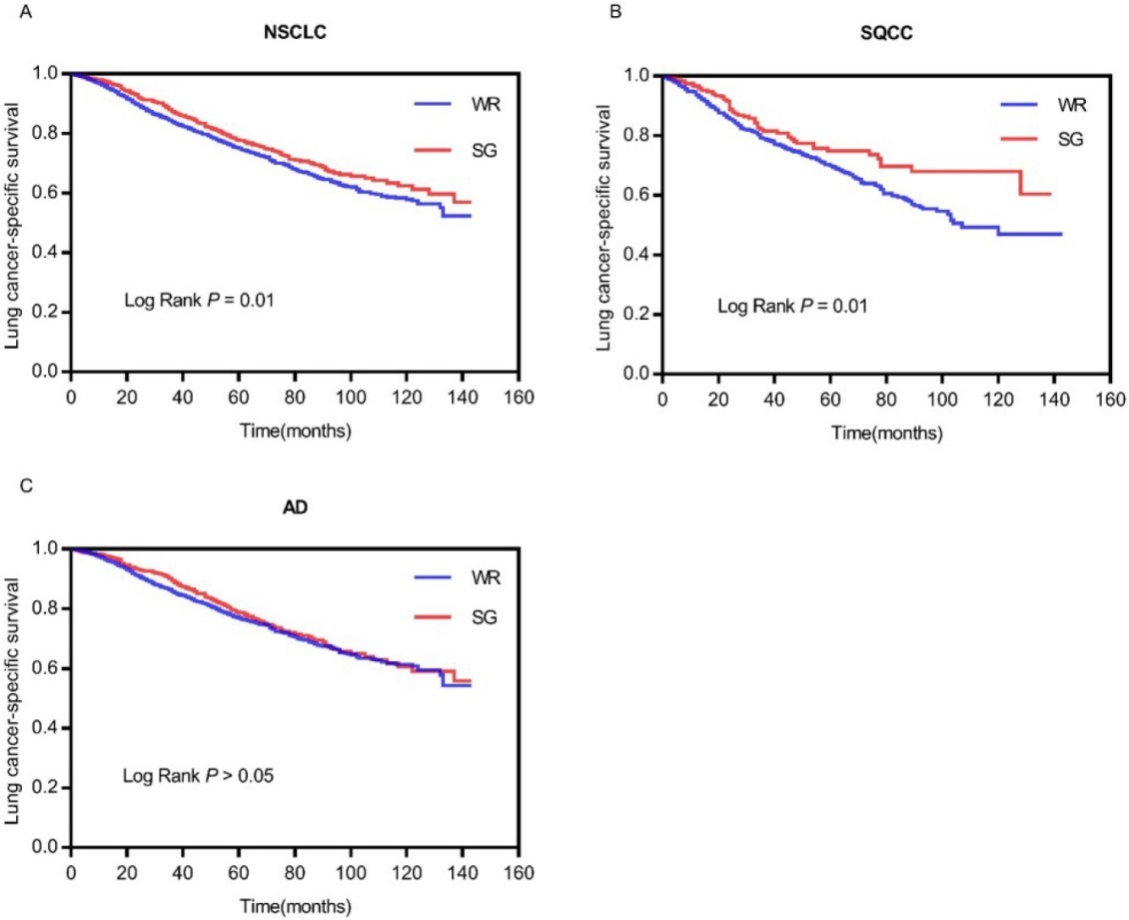
** Comparison of survival curves between WR and SG.** (A) Comparison of survival curves (P=0.01) in patients with stage IA NSCLC; (B) Comparison of survival curves (P=0.01) in patients with stage IA SQCC; (C) Comparison of survival curves (P>0.05) in patients with stage IA AD. Abbreviations: LCSS, lung cancer-specific survival; NSCLC, non-small cell lung cancer; SQCC, squamous cell carcinoma; AD, adenocarcinoma.

According to Kaplan-Meier analysis, the survival curves were compared between WR and SG groups (Figure 2). The survival curve of the SG group is better than the WR group (log rank *P*=0.01) in total patients (Figure 2A). Similarly, the survival curve of the SG group is better (log rank *P*=0.01) than the WR group in patients with stage IA SQCC (Figure 2B). However, in patients with stage IA AD, the survival curves (log rank *P*=0.217) show no statistically significant difference between the WR and SG groups (Figure 2C). This outcome also demonstrates that the SG can yield better survival outcome than WR in patients with stage IA SQCC, but the SG and WR yield generally equivalent survival outcomes in patients with stage IA AD.

### Comparison of lung cancer-specific mortality between the WR and SG groups

The lung cancer-specific mortality was 21.4 (940/4394) for WR group, and 17.8% (214/1205) for SG group in patients with stage IA NSCLC. The lung cancer-specific mortalities were 26.0% (320/1231) and 19.10% (249/1304) for the WR and SG groups in patients with stage IA SQCC, respectively. In patients with stage IA AD, The lung cancer-specific mortalities were 19.6% (620/3163) and 17.3% (157/907) for the WR and SG groups, respectively (Table 4). The outcomes indicate that the lung cancer-specific mortality of SG group is superior to WR group in stage IA SQCC, but this advantage decreases obviously in stage IA AD.

Table 4 also shows the median survival time and the mean survival time of WR and SG groups in different histologic types. In total NSCLC patients, the median survival time and the mean survival time were 38.0 months and 44.56 months for WR group, and 37.00 months and 45.02 months for SG group. In SQCC patients, the median survival time and the mean survival time were 34.00 months and 41.11 months for WR group, and 34.00 months and 44.24 months for SG group. In AD patients, the median survival time and the mean survival time were 39.00 months and 45.90 months for WR group, and 38.00 months and 45.28 months for SG group. The datum demonstrated that the difference of mean survival time is more obvious in SQCC than AD between WR and SG groups.

## Discussion

Currently, with advances in radiology and the emergence of lung cancer screening programs, a higher incidence of early stage NSCLC has been observed. In this scenario, limited resection has been adopted as an effective treatment for stage IA NSCLC patients, especially in the elderly and patients with pulmonary insufficiency. Over the past decade, SG has become more common and played an increasingly important role in the treatment of pulmonary metastases. It is technically difficult for anatomic SG which not only demands a thorough understanding of the pulmonary anatomy, but also requires in-depth tomographic study of the location of the pulmonary nodules. Persistent pneumothorax (over five days) is the most common complication of SG, which occurs between 8% and 10% 19. Therefore, the nodules must be well positioned to ensure safe resection. WR has been considered "inferior" cancer operation for nearly two decades, but much of the contemporary literature is contradictory and inconclusive. Several recent studies have shown that WR is even equivalent to lobectomy 20-22. There is enough controversy to avoid overly dogmatic statements about the poor quality of WR in the absence of modern large-scale prospective trials. Instead, the surgeon should place the emphasis on the quality of the operation and ensure the most possible excision of margin when WR is required. Performed well, WR is maybe an appropriate surgical option.

Some researches has focused on which limited resection was better to the survival of NSCLC patients. Many people have reported that SG is superior to WR for NSCLC patients. For example, Dai et al. 23 considered that SG should be recommended for patients with NSCLC in whom lobectomy is unsuitable. Hou et al. 24 reported that SG results in higher survival rates than WR in patients with stage I NSCLC. Reveliotis et al.^25^ reported that SG is superior to WR in terms of local recurrence and cancer-related mortality rates, and those authors recommended SG for high-risk patients. However, there were some different sounds about this view. In 2016, Altorki et al. 26 reported a retrospective analysis and concluded that WR maybe ontologically equivalent to anatomic SG for clinical T1a tumors. Sybron Harrison et al. 22 has reported a contemporary prospective randomized trial (ACOSOG Z4032) which supported to the view that WR and SG maybe equivalent methods of sub-lobar resection. In our study, we evaluated the survival outcomes of SG versus WR in patients with stage IA NSCLC. Although we found that SG yielded better survival rate than WR in overall patients, there were different in pathology subtypes of NSCLC. The result shows that SG yields better survival rate than WR in patients with stage IA SQCC, but SG and WR yield generally equivalent survival rate in patients with stage IA AD. In addition, patients underwent SG can obtain more advantageous mean survival time than WR in patients with stage IA SQCC, but this advantage almost lost in patients with stage IA AD. This result may provide patients with stage IA AD a personalized surgical option.

Admittedly, there are some limitations in our study, mainly owing to its retrospective design. For example, the lack of original datum from our own studies as well as validation for main findings and conclusion. In addition, the SEER database does not include some important information, such as the types of therapy, histological subtypes, and gene mutations. This information should be included in future prospective studies. Nevertheless, with the inclusion of 12 variables and nearly 5,600 patients in our cohort, the present study represents a well-balanced analysis of SG and WR surgical methods. Thus, in the absence of data from prospective trials, our findings can provide information that is useful for the management of patients with stage IA SQCC and AD.

In summary, the prognosis of patients with stage IA NSCLC is related to a variety of factors.

According to the development trend of modern medicine, there must be a personalized surgical approach which should take into account the characteristics of each patient, the imaging characteristics of tumor as well as the impact on the quality of life and surgical recovery in the future. This study indicates that SG is superior to WR in patients with stage IA SQCC, but the survival outcomes of SG and WR are generally equivalent in patients with stage IA AD. The result may provide a basis for individualized surgical option for stage IA AD patients.

## Figures and Tables

**Table 1:** Baseline characteristics of patients with stage IA non-small cell lung cancer treated with limited resection in the Surveillance, Epidemiology and End Results (SEER) program, 2004-2015

Characteristics	Wedge resection		Segmentectomy		*P*
	Number	%	Number	%	
**Age, year**					<0.001
<45	35	0.8	12	1.0	
≥45, <55	271	6.2	72	6.0	
≥55, <65	950	21.6	276	22.9	
≥65, <75	1734	39.5	487	40.4	
≥75	1404	31.9	358	29.7	
**Sex**					<0.001
Female	2462	56.0	714	59.3	
Male	1932	44.0	491	40.7	
**Tumor size, cm**					<0.001
≤1	893	20.3	167	13.9	
>1, ≤2	2484	56.5	697	57.8	
>2, ≤3	1017	23.2	341	28.3	
**Tumor location**					0.996
Upper lobe	2837	64.5	707	58.6	
Middle lobe	178	4.1	26	2.2	
Lower lobe	1331	30.3	465	38.6	
Not otherwise specified	37	0.8	5	0.4	
Overlapping lesion	11	0.3	2	0.2	
**Differentiation grade**					<0.001
Well differentiated	1054	24.0	278	23.1	
Moderately differentiated	1886	42.9	575	47.7	
Poorly differentiated	1081	24.6	263	21.8	
Undifferentiated	373	8.5	89	7.4	
**Laterality**					0.854
Right-origin of primary	2499	56.9	619	51.4	
Left origin of primary	1895	43.1	586	48.6	
**Histologic type**					<0.001
Adenocarcinoma	3163	72.0	907	75.3	
Squamous cell carcinoma	1231	28.0	298	24.7	
**Radiotherapy**					<0.001
Yes	223	5.1	36	3.0	
No	4143	94.3	1165	96.7	
Others	28	0.6	4	0.3	
**Chemotherapy**					<0.001
Yes	170	3.9	36	3.0	
No	4224	96.1	1169	97.0	
**Marital status**					0.482
Married	2363	53.8	680	56.4	
Single	453	10.3	137	11.4	
Divorced	592	13.5	135	11.2	
Widowed	981	22.3	251	20.8	
Unmarried or domestic partner	5	0.1	2	0.2	
**High school education**					0.079
≥21	793	18.0	244	20.2	
13-20	1304	29.7	279	23.2	
7-12.9	1963	44.7	567	47.1	
<7	334	7.6	115	9.5	

**Table 2:** Multivariate analysis using a cox proportional hazards model in patients with stage IA non-small cell lung cancer

Variable	Multivariate analysis		
	HR	95% CI	*P*
**Sex**			
Female	Reference		
Male	1.296	1.152 to 1.457	<0.001
**Differentiation grade**			<0.001
Well differentiated	Reference		
Moderately differentiated	1.644	1.377 to 1.964	<0.001
Poorly differentiated	1.929	1.592 to 2.339	<0.001
Undifferentiated	1.568	1.216 to 2.023	0.001
**Histologic type**			
Adenocarcinoma	Reference		
Squamous cell carcinoma	1.077	0.945 to 1.228	0.263
**Limited resection**			
Wedge resection	Reference		
Segmentectomy	0.827	0.712 to 0.960	0.013
**Radiotherapy**			<0.001
Yes	Reference		
No	0.648	0.520 to 0.807	<0.001
Others	1.166	0.585 to 2.322	0.662
**Chemotherapy**			
Yes	Reference		
No	0.566	0.452 to 0.709	<0.001
**Tumor size, cm**			<0.001
≤ 1	Reference		
>1, ≤ 2	1.247	1.045 to 1.488	0.014
>2, ≤ 3	1.685	1.393 to 2.039	<0.001
**Age, year**			<0.001
<45	Reference		
≥45, <55	1.892	0.757 to 4.728	0.172
≥55, <65	1.875	0.772 to 4.554	0.165
≥65, <75	2.048	0.846 to 4.959	0.112
≥75	2.975	1.228 to 7.208	0.016

Abbreviations: HR, hazard ratio; CI, confidence interval.

**Table 3:** Univariate analysis comparing HR (SG vs. WR) in patients

Variable	Number	Univariate Analysis		
		HR	95% CI	*P*
Total	5599	0.823	0.710 to 0.955	0.010
SQCC	1529	0.689	0.519 to 0.914	0.010
AD	4070	0.896	0.752 to 1.067	0.217

Abbreviations: SQCC, squamous cell carcinoma; AD, adenocarcinoma; SG, segmentectomy; WR, wedge resection; HR, hazard ratio; CI, confidence interval.

**Table 4:** Lung cancer-specific mortality, median survival time and mean survival time of WR and SG in different histologic types (SEER database, 2004-2015)

Histologic type	Mortality n/N (%)		Median survival time (months)		Mean survival time (months)	
	WR	SG	WR	SG	WR	SG
NSCLC	21.4 (940/4394)	17.8 (214/1205)	38.00	37.00	44.56	45.02
SQCC	26.0 (320/1231)	19.1 (57/298)	34.00	34.00	41.11	44.24
AD	19.6 (620/3163)	17.3 (157/907)	39.00	38.00	45.90	45.28

Abbreviations: NSCLC, non-small cell lung cancer; SQCC, squamous cell carcinoma; AD, adenocarcinoma; WR, wedge resection; SG, segmentectomy.

## Abbreviations

NSCLC: non-small cell lung cancer
WR: wedge resection
SG: segmentectomy
SEER: Surveillance, Epidemiology and End Results
LCSS: lung cancer-specific survival
SQCC: squamous cell carcinoma
AD: adenocarcinoma
NOS: not otherwise specified
HR: hazard ratio
CI: confidence interval
